# The effect of knee joint rotation in the sagittal and axial plane on the measurement accuracy of coronal alignment of the lower limb

**DOI:** 10.1186/s12891-020-03487-9

**Published:** 2020-07-17

**Authors:** Hyun-Soo Moon, Chong-Hyuk Choi, Min Jung, Dae-Young Lee, Jung-Hwan Kim, Sung-Hwan Kim

**Affiliations:** 1 Arthroscopy and Joint Research Institute, Yonsei University College of Medicine, Seoul, Republic of Korea; 2 Department of Orthopedic Surgery, Hallym University Sacred Heart Hospital, Hallym University College of Medicine, Anyang, Republic of Korea; 3 Department of Orthopedic Surgery, Severance Hospital, Yonsei University College of Medicine, Seoul, Republic of Korea; 4 Department of Orthopedic Surgery, Saegil Hospital, Seoul, Republic of Korea; 5 Department of Orthopedic Surgery, Gangnam Severance Hospital, Yonsei University College of Medicine, Seoul, Republic of Korea

**Keywords:** Knee joint rotation, Measurement accuracy, Coronal alignment of the lower limb, EOS image

## Abstract

**Background:**

Although the measurement of coronal alignment of the lower limb on conventional full-length weight-bearing anteroposterior (FLWAP) radiographs was reported to be influenced by the knee joint rotation, no comparative analysis was performed considering the effects of knee joint rotation on the sagittal and axial planes simultaneously using the three-dimensional images while taking into account the actual weight-bearing conditions. The aim of this study was to investigate the effect of knee joint rotation on the measurement accuracy of coronal alignment of the lower limb on the FLWAP radiograph.

**Methods:**

Radiographic images of 90 consecutive patients (180 lower limbs) who took both the FLWAP radiograph and the EOS image were retrospectively reviewed. The relationship among delta values of mechanical tibiofemoral angle (mTFA) between the FLWAP radiographs and the EOS images (ΔmTFA), knee flexion/extension angle (sagittal plane rotation) on the EOS images, and patellar rotation (axial plane rotation) on the FLWAP radiographs were analyzed. Further, subgroup analysis according to each direction of knee joint rotation was performed.

**Results:**

There was a significant correlation between ΔmTFA and sagittal plane rotation (*r* = 0.368, *P* <  0.001), whereas axial plane rotation was not correlated. In the analysis according to the direction, statistically significant correlation was observed only in the knee flexion group (*r* = 0.399, *P* <  0.001). The regression analysis showed a significant linear relationship between ΔmTFA and sagittal plane rotation (*r*^*2*^ = 0.136, *P* <  0.001). Additional subgroup analysis in patients with the patellar rotation greater than 3% showed a similar result of a linear relationship between ΔmTFA and sagittal plane rotation (*r*^*2*^ = 0.257, *P* <  0.001), whereas no statistically significant relationship was found in patients with the patellar rotation less than 3%.

**Conclusion:**

The measurement accuracy of coronal alignment of the lower limb on the FLWAP radiographs would be influenced by knee flexion, specifically when there is any subtle rotation of the knee joint in the axial plane. A strict patellar forward position without axial plane rotation of the knee could provide accurate results of the measurement even if there is a fixed flexion contracture of the knee.

## Background

Accurate measurement of the lower limb alignment is highly important for patient management in the orthopedic practice, especially in osteotomy surgery and joint replacement surgery [1, 2]. It is essential not only for the preoperative surgical planning but also for the postoperative evaluation. Of those, measurement of coronal alignment would be the most crucial factor [3], which enables an assessment through various radiographic parameters such as mechanical axis deviation, mechanical tibiofemoral angle (mTFA), joint convergence angle, and etc.

In clinical practice, most measurements for coronal alignment of the lower limb have been made with the full-length weight-bearing anteroposterior (FLWAP) radiographs of both lower extremities, which is a conventional method obtained from two-dimensional (2D) projection. However, it has been reported that the measurement of the lower limb alignment with 2D image could be influenced by knee joint rotation in the sagittal plane as well as the axial plane [4–6]. Considering the fact that most knees with osteoarthritis are frequently associated with the flexion contracture as well as the rotational deformity, it is essential to take into account the effect of rotation of the knee when evaluating coronal alignment of the lower limb [7, 8]. Inaccurate assessment of coronal alignment of the lower limb owing to the abovementioned factors would lead to improper management of the patients.

To overcome these problems, several three-dimensional (3D) imaging modalities such as computed tomography (CT), intraoperative navigation system, and magnetic resonance image (MRI) were utilized to enhance the accuracy in the measurement of the lower limb alignment [9–11]. However, these modalities did not accurately reflect the actual weight-bearing condition, which subsequently could cause a potential error in the evaluation of the limb alignment [12–16]. Furthermore, the problems of unnecessary radiation exposure and cost-effectiveness could also be raised [17]. In this regard, the EOS imaging system (EOS® imaging inc, Paris, France), which simultaneously provides a biplanar image of the lower limb and enables the 3D reconstruction, has been suggested as a good alternative imaging modality. Since it not only provides 3D information of the lower limbs with a low radiation dose but it also allows weight-bearing condition [17–19], it could reflect the actual state of the lower limb alignment more accurately than pre-existing evaluation modalities. Therefore, based on the measurement values of the lower limb alignment obtained from the EOS images, it is possible to evaluate the effect of the knee joint rotation on the measurement accuracy of lower limb alignment on the conventional FLWAP images. To the author’s best knowledge, no comparative analysis was performed considering the effects of knee joint rotation on the sagittal and axial planes simultaneously using the three-dimensional images while taking into account the actual weight-bearing conditions.

The purpose of the present study was to investigate the effect of knee joint rotation in the sagittal plane and axial plane to the measurement accuracy of coronal alignment of the lower limb on the FLWAP radiograph with reference to the values measured by the EOS system. The hypothesis was that the axial plane rotation, rather than sagittal plane rotation, would affect the measurement accuracy of coronal alignment of the lower limb.

## Methods

### Subject enrollment

**Fig. 1:**
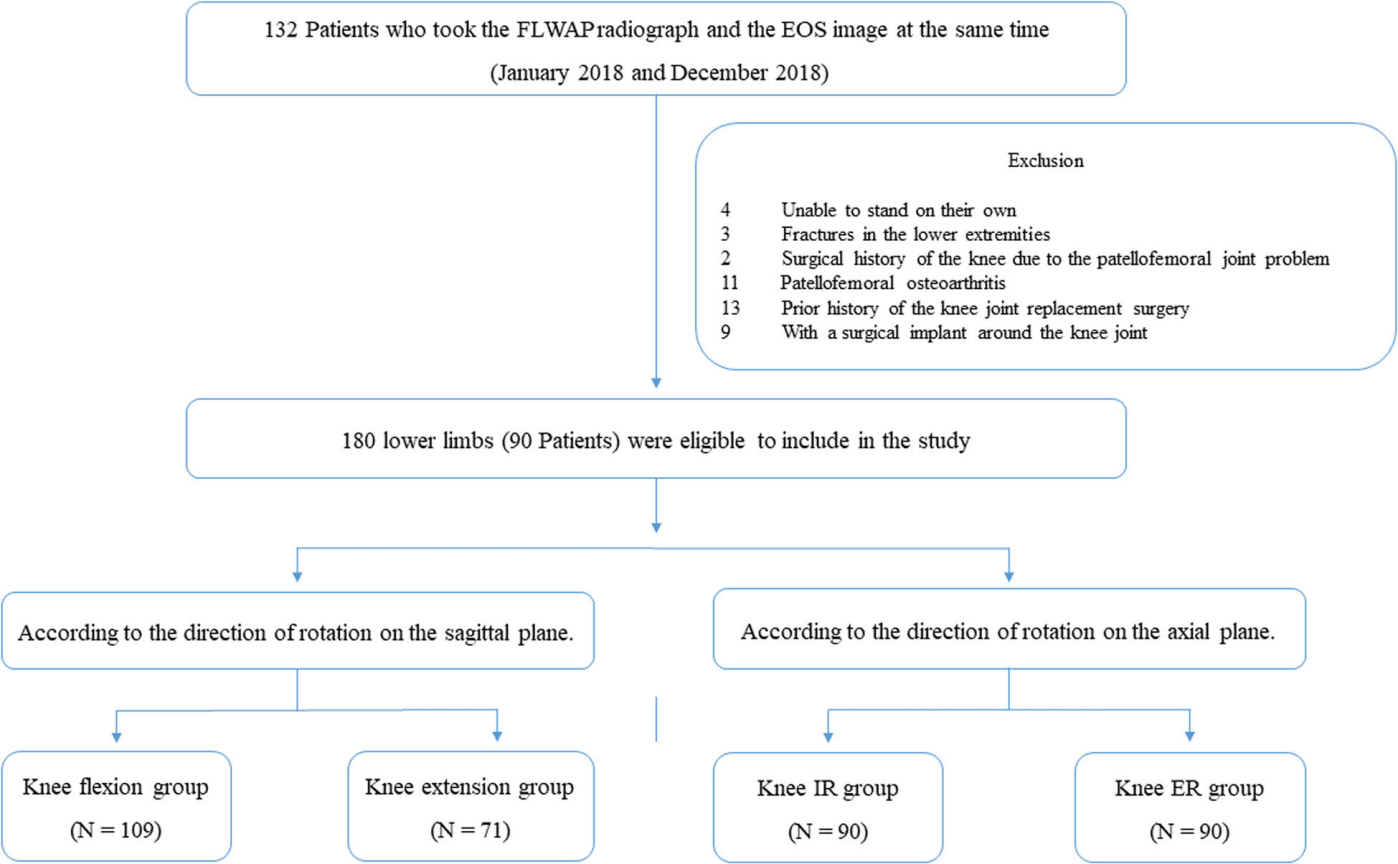
Flowchart of patients included in this study

The present study was approved by the Institutional Review Board (ID Number: 3–2019-0253), which waived the requirement for informed consent from the patients owing to the retrospective nature of the study. Between January 2018 and December 2018, the data of the consecutive patients who visited the orthopedic outpatient clinics in our institution were retrospectively reviewed. Of those, the patients who had taken the FLWAP radiograph and the EOS image at the same time were included in this study. The exclusion criteria were as follows: (1) patients who were unable to stand on their own at the time of the evaluation; (2) patients with fractures in the lower extremities; (3) patients with patellofemoral osteoarthritis; (4) patients with a surgical history of the knee due to the patellofemoral joint problem; (5) patients who had previously undergone knee joint replacement surgery; (6) patients with a surgical implant around the knee joint. Ninety patients (the mean age: 48.0 ± 16.5 years, 47 males and 43 females), a total of 180 lower limbs, were eligible to include in the study. Subsequently, according to the direction of knee joint rotation on each of the sagittal and the axial plane, subjects were classified into two groups on the sagittal plane (knee flexion group and knee extension group) and also divided into two groups on the axial plane (knee internal rotation group and knee external rotation group). As a result, four subgroups were analyzed in the present study (Fig. 1).

### Radiographic assessment

The FLWAP radiograph was taken for all patients who complained of knee pain to assess coronal alignment of the lower limb in our institution. Of those, an additional EOS image was taken for the patients who needed an evaluation of the rotation on the axial plane of the lower extremity on behalf of CT scan. The FLWAP radiograph of both lower extremities was taken with the patient standing in weight-bearing condition facing forward the x-ray tube (90 Kvp [kilovoltage], 50mAs [milliampere seconds], FFD [focus-to-film distance] of 260 cm, 3 acquired images). The images were taken with the Philips DigitalDiagnost x-ray system (Philips Healthcare Inc.), and the software automatically stitches acquired images into one composite image. The patients were led to load full their weight on both lower extremities equally without any assisted device. In addition, they were instructed to keep their knees not to flex intentionally and were adjusted with patella facing forward the x-ray tube. The EOS image was taken with the EOS® Imaging device, which is consisted of two co-linked pairs of the x-ray tubes and the corresponding detectors. These co-linked units are placed perpendicular to each other in the frontal and lateral side within the apparatus, which enables to capture of the bi-planar x-ray image simultaneously. The synchronized vertical movement of the paired units allows covering a wide area of 170 cm high and 45 cm wide, while a fraction of radiation exposure was 800 to 1000 times lower than the CT scan [18]. Patients were instructed to be in the same condition as in the FLWAP radiograph during the evaluation by placing the load equally on both lower extremities, not to flex knees, and with the patella facing forward. Simultaneously obtained 2D images of the lower extremities were reconstructed to the 3D image using the sterEOS workstation (EOS® Imaging Inc., Paris, France). The reconstruction process was based on the software-guided procedure, which has previously been validated for the evaluation of the rotational alignment of the lower extremity [20, 21]. 3D models of the lower extremities are semi-automatically adapted to the osseous contour of the femur and tibia, respectively, on bi-planar x-ray images. During this fitting process, several anatomical reference points were identified precisely by spatial manipulation of the object, using the epipolar line which makes it possible to simultaneously reflect each reference points on the coronal and the sagittal plane. The plane connecting the center of the femoral head and the transcondylar line (the axis with the minimal mediolateral patellar shift, connecting the center of both femoral condyles) was defined as the frontal plane [22], which was considered as an anterior-posterior view on the EOS image for the assessment of the coronal alignment. The sagittal plane, a lateral view for the evaluation of sagittal alignment, was automatically set to the plane orthogonal to the frontal plane. Based on these anatomical reference points, several radiographic parameters are automatically calculated and recorded.

### Measurement of the lower limb alignment

**Fig. 2:**
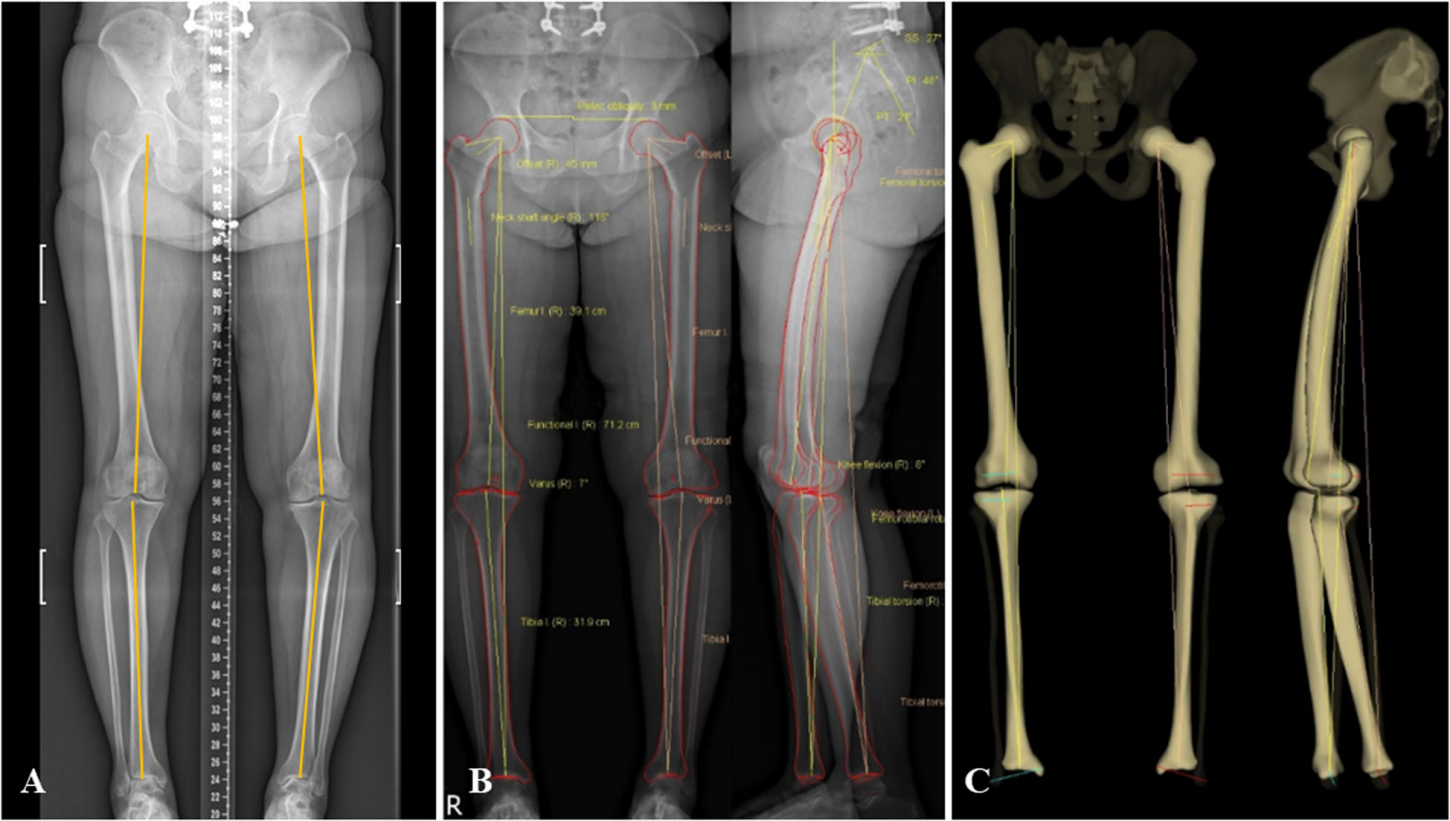
Example of the FLWAP image and the EOS image in the same patient. The images were taken in the same position with the knee not to flex intentionally and the patella facing forward. The mTFA on the FLWAP image was represented as a yellow line in (**a**), and the mTFA on the EOS image was semiautomatically displayed (**b**, **c**). *FLWAP* full-length weight-bearing anteroposterior, *mTFA* mechanical tibio-femoral angle

**Fig. 3:**
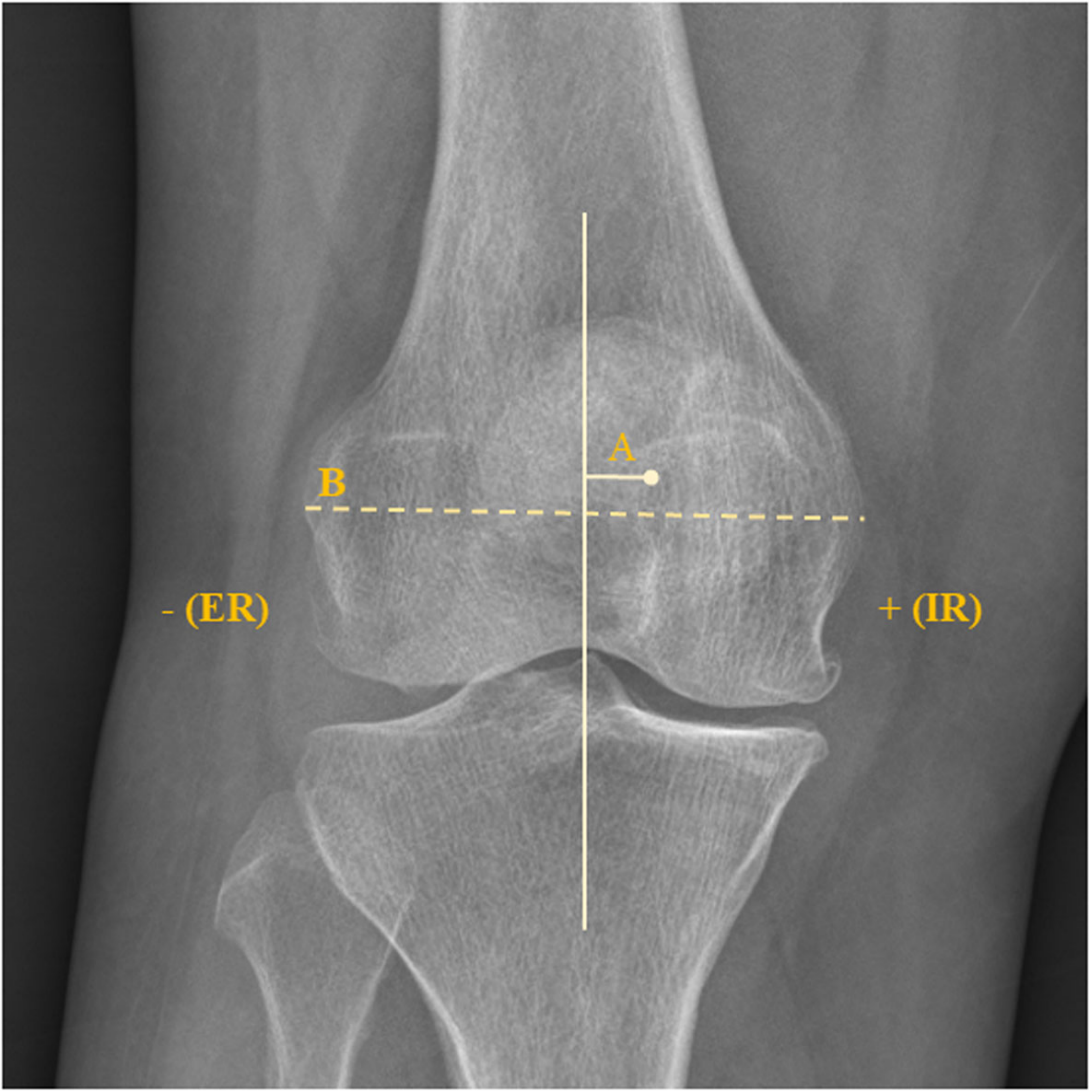
The measurement method of the axial plane rotation of the knee in this study. It was defined as the degree of deviation of the patellar center inward or outward relative to the midpoint of the line connecting both femoral epicondyles (A/B * 100, %)

Coronal alignment of the lower limb was measured with the mTFA in both FLWAP radiographs and EOS images. The mTFA was defined as an acute angle formed by the femoral mechanical axis and the tibial mechanical axis [23], in which varus alignment was set to have a positive value and valgus alignment was set to have a negative value in the current study. The mechanical axis of the femur was defined as the line connecting the center of the femoral head and the center of the femoral intercondylar notch, and the mechanical axis of the tibia was defined as the line connecting the center of the tibial plateau and the center of distal articular surface of the tibia (Fig. 2a). The sagittal alignment of the lower limbs, a sagittal plane rotation of the knee joint described as a knee flexion/extension angle, was measured on the EOS images. It was defined as the angle between the femoral mechanical axis and the tibial mechanical axis in the sagittal femoral plane. The values at knee flexion were set as a positive value, and the values at knee extension were set as a negative value. The classification between these two groups was based on 0 degrees of knee flexion/extension angle. The coronal and sagittal alignment of the lower limb on the EOS images are semi-automatically defined based on the previously determined several anatomical reference points during the process of 3D model reconstruction (Fig. 2b, c). The axial plane rotation of the knee joint, described as a patellar rotation in this study, was assessed on the FLWAP radiographs. This was defined as the position of the patella with respect to the femoral condyles, in which the amount of rotation was determined by the degree of deviation of the patellar center inward or outward relative to the midpoint of the line connecting the medial and lateral epicondyle (Fig. 3). It was set to have a positive value when the patella rotated internally, and a negative value when the patella rotated externally, and their classification was based on the direction of the patellar center compared to the midpoint of the line connecting both femoral epicondyles. Based on the radiographic parameters mentioned above, the relationship between the mTFA (ΔmTFA), the difference between the mTFA on the FLWAP radiograph and the EOS images, and the degree of rotation in the sagittal and the axial planes were investigated.

To assess the inter-rater reliability, radiographic measurements on the FLWAP image were assessed by two orthopedic surgeons who were blinded to patient information with an interval of 6 weeks, using a picture archiving and communication system (GE Medical System Information Technologies). For the EOS image, 3D model reconstruction was performed by an independent radiology technician who is trained to manage the sterEOS software.

### Statistical analysis

Prior to the study, the statistical power was calculated using G*POWER software version 3.1.9.2 (Franz et al., Universitat Kiel, Germany). The estimated correlation coefficient was set at 0.334, which was obtained from a preliminary correlation analysis with 30 subjects (60 lower limbs). With the significance level (alpha) of 5% and power (1-beta) of 80%, the minimum sample size required for the present study was revealed to be 68.

All other statistical analysis was performed using IBM SPSS statistics version 25.0 (IBM Corp., Armonk, NY, USA). A paired *t*-test was used to compare ΔmTFA on the FLWAP radiographs and on the EOS images. The Pearson correlation analysis and the partial correlation analysis were conducted to evaluate the association between the ΔmTFA and the rotation of the knee joint in the sagittal plane and the axial plane. In addition, a linear regression analysis was performed to identify the relationship in terms of the dependencies. The analyses for the association between variables were based on the absolute value. Since values in different directions (positive or negative values) might counterbalance each other during the analytic process and affect the results, the influence of the direction of rotation (e.g. flexion or extension) was assessed by dividing the groups in each direction of knee joint rotation. Accordingly, the effect of the degree of rotation on each plane, as well as the direction of rotation, were analyzed comprehensively. The intra-rater and inter-rater reliabilities were calculated using the intraclass correlation coefficient (ICC) set at a 95% confidence interval. The level of significance was set at *P* <  0.05.

## Results

The number of subjects classified according to the direction of knee joint rotation on each plane was as follows: knee flexion group (*N* = 109) and extension group (*N* = 71), or knee internal rotation group (*N* = 90) and external rotation group (*N* = 90). There was a significant difference between the mTFA on the FLWAP radiographs and on the EOS image (*P* <  0.001) (Table 1). The mean value of absolute ΔmTFA, knee flexion/extension angle, and patellar rotation in overall subjects was 1.7 ± 2.0°, 5.3 ± 5.3°, 4.6 ± 4.0%, respectively (Table 1).

**Table 1:** Baseline radiographic parameters of the subjects

Baseline radiographic parameter	value^*^
mTFA (°)	
On FLWAP radiograph	1.2 ± 3.9
On EOS image	2.1 ± 4.1
ΔmTFA (°)^†^	1.7 ± 2.0
Sagittal plane rotation (Knee flex/ext. Angle, °)	
Overall cohort (*N* = 180)^†^	5.3 ± 5.3
Knee flexion group (*N* = 109)	6.0 ± 6.3
Knee extension group (*N* = 71)	−4.4 ± 3.2
Axial plane rotation (Patellar rotation, %)	
Overall cohort (*N* = 180)^†^	4.6 ± 4.0
Knee internal rotation group (*N* = 90)	4.1 ± 3.5
Knee external rotation group (*N* = 90)	−5.1 ± 4.4

*The values are given as means and standard deviations

†The values are analyzed and presented based on absolute value

*mTFA* mechanical tibio-femoral angle; *FLWAP* full-length weight-bearing anteroposterior; *ΔmTFA* absolute value of delta mechanical tibio-femoral angle; *flex/ext*. Flexion/Extension

The Pearson correlation analysis was performed to evaluate the association between variables. There was significant correlation between the ΔmTFA and sagittal plane rotation of the knee (*r* = 0.368, *P* <  0.001), while there was no correlation between the ΔmTFA and axial plane rotation of the knee (Table 2). In the analysis according to the direction, there was a significant correlation with ΔmTFA only in the knee flexion group (*r* = 0.399, *P* <  0.001) (Table 2). Similar trends were observed in the partial correlation analysis considering the effect of the rotation of the other plane. There was a statistically significant correlation between the ΔmTFA and the sagittal plane rotation of the knee (*r* = 0.369, *P* <  0.001), especially in the knee flexion group (*r* = 0.403, *P* <  0.001) (Table 3).

**Table 2:** Results of linear correlation analysis between the variables

	*r* value^*^	*P* value
ΔmTFA and sagittal plane rotation of the knee^†^		
Overall cohort (*N* = 180)	0.368	< 0.001
Knee flexion group (*N* = 109)	0.399	< 0.001
Knee extension group (*N* = 71)	−0.139	n.s.
Knee internal rotation group (*N* = 90)	0.644	< 0.001
Knee external rotation group (*N* = 90)	0.210	0.047
ΔmTFA and axial plane rotation of the knee^†^		
Overall cohort (*N* = 180)	0.018	n.s.
Knee flexion group (*N* = 109)	0.075	n.s.
Knee extension group (*N* = 71)	−0.111	n.s.
Knee internal rotation group (*N* = 90)	0.075	n.s.
Knee external rotation group (*N* = 90)	−0.011	n.s.
Sagittal plane rotation and axial plane rotation of the knee^†^		
Overall cohort (*N* = 180)	−0.015	n.s.
Knee flexion group (*N* = 109)	−0.036	n.s.
Knee extension group (*N* = 71)	0.059	n.s.
Knee internal rotation group (*N* = 90)	0.027	n.s.
Knee external rotation group (*N* = 90)	−0.05	n.s.

*The correlation coefficient

†The analyses were based on absolute value

ΔmTFA, absolute value of delta mechanical tibio-femoral angle

**Table 3:** Results of partial correlation analysis between the variables

	*r* value^*^	*P* value
ΔmTFA and sagittal plane rotation of the knee^†^		
Overall cohort (*N* = 180)	0.369	< 0.001
Knee flexion group (*N* = 109)	0.403	< 0.001
Knee extension group (*N* = 71)	0.147	n.s.
ΔmTFA and axial plane rotation of the knee^†^		
Overall cohort (*N* = 180)	0.026	n.s.
Knee internal rotation group (*N* = 90)	0.075	n.s.
Knee external rotation group (*N* = 90)	0.000	n.s.

*The correlation coefficient

†The analyses were based on absolute value

ΔmTFA, absolute value of delta mechanical tibio-femoral angle

**Fig. 4:**
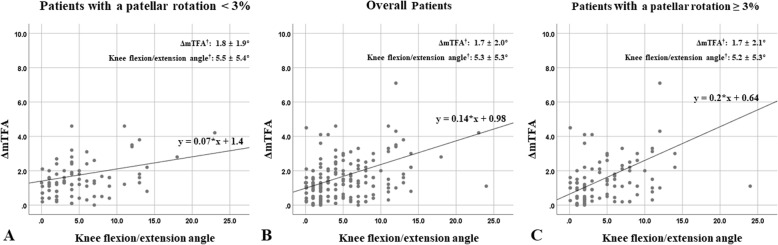
Scatter graphs are shown of a linear relationship between the ΔmTFA and the sagittal plane rotation of the knee. The analysis was performed in patients with a patellar rotation less than 3% (*r*^2^ = 0.039, *P* = 0.074) (**a**), in overall patients (*r*^2^ = 0.136, *P* < 0.001) (**b**), and in patients with a patellar rotation greater than 3% (*r*^2^ = 0.257, *P* < 0.001) (**c**), respectively. *ΔmTFA* delta value of the mechanical tibio-femoral angle, *r*^*2*^ the coefficient of determination. † The values are given as means and standard deviations, analyzed based on absolute value

**Fig. 5:**
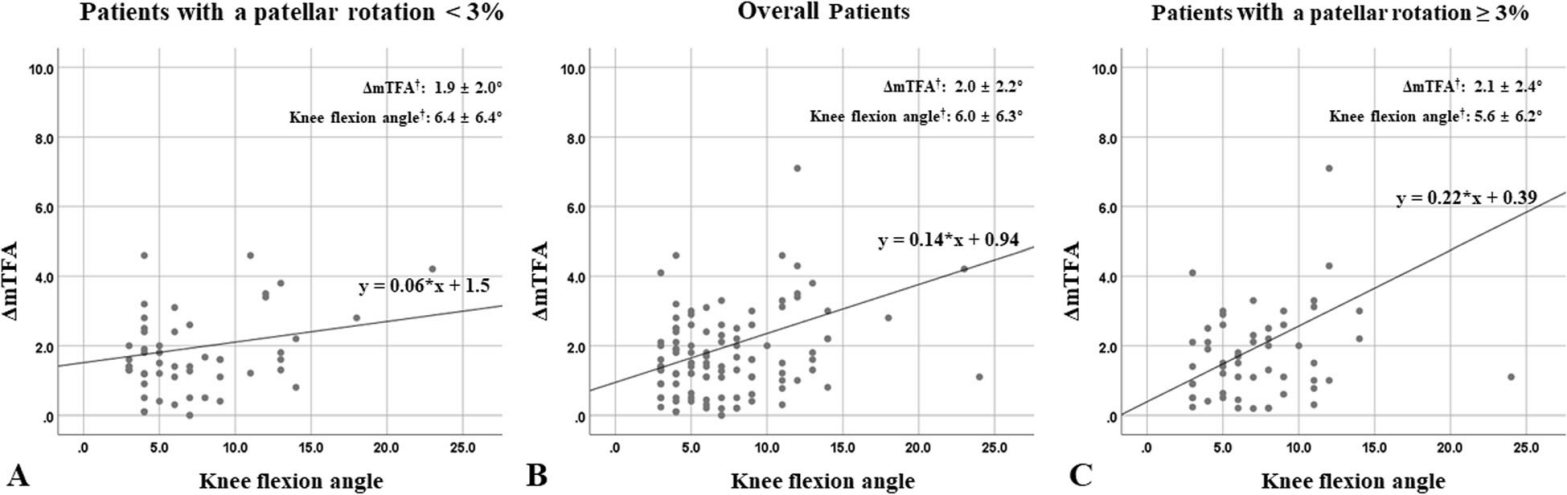
Scatter graphs of the knee flexion group are shown of a linear relationship between the ΔmTFA and the sagittal plane rotation of the knee. The analysis was performed in patients with a patellar rotation less than 3% (*r*^2^ = 0.028, *P* = 0.221) (**a**), in overall patients (*r*^2^ = 0.121, *P* < 0.001) (**b**), and in patients with a patellar rotation greater than 3% (*r*^2^ = 0.236, *P* < 0.001) (**c**), respectively. † The values are given as means and standard deviations, analyzed based on absolute value

Since there was a correlation between the ΔmTFA and the sagittal plane rotation of the knee, a univariate linear regression analysis was performed to evaluate the relationship in terms of dependency between them, resulting in a significant linear relationship between the two variables (*r*^*2*^ = 0.136, *P* < 0.001) (Fig. 4b). At this point, we performed an additional analysis of the relationship between the ΔmTFA and the sagittal plane rotation of the knee according to the degree of patellar rotation. Since the FLWAP radiographs had been made under the precondition of the patellar forward position in the enrolled subjects, this could subsequently not fully reflect the effect of the axial plane rotation of the knee. Therefore, based on the reference value suggested by the previous study [6], an additional univariate regression analysis was performed, divided into patients with the patellar rotation greater than 3% (*N* = 97) and the patellar rotation less than 3% (*N* = 83). It revealed that there was a significant linear relationship between the ΔmTFA and the sagittal plane rotation of the knee in patients with patellar rotation greater than 3% (*r*^*2*^ = 0.257, *P* < 0.001), whereas there was no statistically significant relationship in patients with patellar rotation less than 3% (Fig. 4a, c). Similar results were also observed when the knee flexion group was analyzed with a univariate linear regression model in the same fashion as before (Fig. 5).

The 95% confidence intervals for ICCs were 0.94 to 0.98 (observer 1) and 0.91 to 0.96 (observer 2) for intra-observer reliabilities and were 0.84 to 0.92 for inter-observer reliabilities.

## Discussion

The principal finding of the current study was that the measurement accuracy of coronal alignment of the lower limb on the FLWAP radiograph was influenced by knee flexion, which was significant when there was any subtle rotation of the knee joint in the axial plane. To improve the accuracy of the measurement, it would be essential to check whether the patella is rotated, especially in patients with knee flexion.

Accurate measurement of coronal alignment of the lower limb is one of the most crucial factors in the patient evaluation and treatment in the clinical practice. Since just a few degrees of difference in the lower limb alignment can affect clinical outcomes during the orthopedic surgical procedure such as knee joint replacement surgery and osteotomy procedure, precise assessment of the lower limb alignment is essential [1, 24, 25]. Most of the assessments of coronal alignment of the lower limb have been made with the FLWAP radiographs, however, it is reported that the measurement accuracy of the lower limb alignment could be affected by the knee joint rotation [4–6]. Although 3D imaging modalities such as CT scan, intraoperative navigation system and MRI were utilized to overcome this drawback raised from 2D image, it has been pointed out that these 3D imaging techniques could not reflect an actual weight-bearing condition of the patient [12–16]. As the EOS imaging system reflects the actual weight-bearing condition and presents three-dimensional information, it could provide reliable information about the lower limb alignment. However, the EOS imaging system could not be available in all institution and the measurement of the lower limb alignment on the FLWAP radiograph has been regarded as a standardized way so far. Therefore, it is reasonable to study how to measure more accurately the lower limb alignment on the FLWAP radiograph by investigating the relevant factors affecting the accuracy of the measurement.

According to the present study, there was a significant association between the ΔmTFA and the sagittal plane rotation of the knee, especially in the knee flexion. However, even though the univariate linear regression analysis showed linear relationship between these variables, an analysis in subjects with the patellar rotation less than 3% did not show statistically significant results. On the contrary, in the analysis of patients with patellar rotation greater than 3%, the strength of the relationship between variables was higher than in the analysis with the overall subjects. This can be interpreted as that even if there is some rotation of the knee joint on the sagittal plane, such as knee flexion or extension, it would not affect the measurement accuracy of coronal alignment of the lower limb when the patella is precisely facing forward without any rotation on the axial plane. Although no significant association was found between patellar rotation and the ΔmTFA or the sagittal plane rotation of the knee in the correlation analysis, this is considered to result from the fact that all utilized images in the study were taken under the condition of patellar forward, which subsequently could not fully reflect the effect of axial plane rotation of the knee joint. The result of the current study is in line with those of the preceding studies [4, 6]. According to the recent study of Shetty et al., flexion deformity of the knee > 10° would significantly affect the interpretation of the coronal mechanical alignment [4]. In regard to the axial plane rotation, it was reported that even with a 3° of axial plane rotation of the lower extremity would cause an error in the evaluation of the coronal alignment of the lower limb [6]. Although these studies provided us valuable information regarding the effects of the rotation on the reliability of the measurement of coronal alignment of the lower limb, the effects in both sagittal and axial planes were not considered at the same time. Therefore, the present study is meaningful in that it comprehensively considered the effects of each plane simultaneously. Furthermore, since it is reported that the measurement of the lower limb alignment values could vary up to 2.5° depending on the weight-bearing status [16], this study has the strengths in that it reflected actual weight-bearing conditions in all images.

The present study revealed that knee joint rotation would affect the measurement accuracy of coronal alignment of the lower limb. Accordingly, great caution should be taken in the process of taking the FLWAP radiograph as well as in the measurement as this can lead to misinterpretation of the results, which in turn could affect the diagnostic and therapeutic management of patients. The image should be obtained with the knee not to be flexed as possible. Furthermore, even if there is a fixed flexion contracture of the knee that could not be controlled, a strict patellar forward position without axial plane rotation of the knee could provide a more accurate result of the measurement.

This study has several limitations. First, the present study is retrospective in nature, which could be associated with the risk of bias in evaluation. Second, various radiographic parameters such as medial proximal tibial angle, lateral distal femoral angle, and joint convergence angle were not evaluated. Third, the degree of knee joint rotation in the sagittal plane cannot be exactly the same in the FLWAP radiographs and in the EOS images. Although patients were instructed not to flex the knees intentionally during each assessment, there might be subtle differences in the knee flexion/extension angle between two images. Likewise, the weight-bearing status could also be different. Fourth, as mentioned above, since all images were taken with the condition that the patella was facing forward, the degree of patella rotation was basically small. This subsequently could not fully reflect the effects of the axial plane rotation of the knee joint. Further, even though patients with patellofemoral problems were excluded prior to the study, there may be unrecognized factors that might affect the patellofemoral alignment.

## Conclusions

The measurement accuracy of coronal alignment of lower limb on the FLWAP radiographs would be influenced by knee flexion, specifically when there is any subtle rotation of the knee joint in the axial plane. A strict patellar forward position without axial plane rotation of the knee could provide accurate results of the measurement even if there is a fixed flexion contracture of the knee.

## Data Availability

The datasets used and/or analyzed in this study available from the corresponding author on reasonable request.

## Abbreviations

mTFA: Mechanical tibiofemoral angle
FLWAP: Full-length weight-bearing anteroposterior
2D: Two-dimensional
3D: Three-dimensional
CT: Computed tomography
MRI: Magnetic resonance image
Kvp: Kilovoltage
mAs: Milliampere seconds
FFD: Focus-to-film distance
ΔmTFA: Absolute value of delta mechanical tibio-femoral angle
ICC: Intraclass correlation coefficient
